# Comprehensive Assessment of Corvis ST Biomechanical Indices in Normal and Keratoconus Corneas with Reference to Corneal Enantiomorphism

**DOI:** 10.3390/jcm12020690

**Published:** 2023-01-15

**Authors:** Vincent Borderie, Juliette Beauruel, Roxane Cuyaubère, Cristina Georgeon, Benjamin Memmi, Otman Sandali

**Affiliations:** GRC 32, Transplantation et Thérapies Innovantes de la Cornée, Centre Hospitalier National d’Ophtalmologie des Quinze-Vingts, Sorbonne Université, 75006 Paris, France

**Keywords:** biomechanics, cornea, enantiomorphism, keratoconus

## Abstract

The aim of this study was to assess Corvis ST biomechanical indices in reference to corneal enantiomorphism. In a prospective observational cohort study, 117 eyes from 63 patients with normal or keratoconus corneas were assessed by three independent observers. In the control group (n = 62), no significant differences were observed between the three observers for all indices. The best reproducibility was obtained with pachymetry and the weakest with CBI. All indices but CBI and arc length featured COV < 10%. All indices except the PD and SSI correlated with pachymetry; all but Rad correlated with IOP. The comparison of the thinnest with the thickest corneas showed no significant differences for any index except pachymetry. In the keratoconus group (n = 55), loss of corneal enantiomorphism was confirmed for all indices except the arc length, velocity, and PD. Significant differences between both groups were found for all indices, even after adjustment for pachymetry and intraocular pressure. The CBI featured the best accuracy (92%), sensitivity (91%), and graphical relevance for keratoconus diagnosis. However, its reproducibility was weak in normal corneas and was strongly dependent on corneal thickness. The SSI was independent of corneal thickness, highly reproducible, and provided the expected enantiomorphism characteristics in both groups, making it a relevant biomarker of biomechanical corneal behavior.

## 1. Introduction

The cornea exhibits a viscoelastic behavior that is crucial for maintaining its curvature and subsequent refractive power despite changes in intraocular pressure and various forces, such as external shocks or eye-rubbing. Corneal biomechanical properties are closely related to the structure of stroma, which consists of several hundred 1–3 µm-thick stacked lamellae made of collagen fibrils that are aligned and regularly packed. This corneal viscoelastic behavior is explained by the rearrangement and sliding of the stromal lamellae and the stretching of the stromal striae during stress [1].

Keratoconus is a bilateral progressive corneal disease characterized by focal biomechanical weakening, localized conical protrusion, apical thinning, and irregular astigmatism. Areas outside the conus feature normal biomechanical properties. The finite element model of keratoconus shows eccentric thinning and a reduced modulus [2]. Specific changes in the corneal stroma structure are associated with alterations in the viscous and elastic properties of the keratoconus cornea [3]. The reported incidence of keratoconus is much higher among candidates for refractive surgery [4]. Preoperative accurate detection of keratoconus among refractive surgery candidates is crucial because refractive surgery might be contraindicated in these patients.

Corneal biomechanical behavior can be assessed with various technologies, including strip extensometry, eye inflation, Brillouin microscopy, air-puff systems, ultrasound elastography, optical coherence tomography elastography, enzymatic digestion, and laser interferometry [5]. In routine practice, only air-puff systems are currently available for the clinical assessment of corneal biomechanical behavior [6]; they provide a global evaluation of corneal biomechanics. The Ocular Response Analyzer device is the first widely used air-puff device that provides an assessment of corneal hysteresis. Its limitations include its weak reproducibility and the high variability of its measurements [7]. In addition, hysteresis is highly dependent on corneal central thickness, so it cannot be used alone to identify keratoconus suspect corneas [8,9]. The corneal visualization Scheimpflug technology (Corvis ST) device analysis method is based on a corneal dynamic deformation video that is recorded using Scheimpflug technology [10]; it has been reported to provide an analysis of corneal deformation independent of central corneal thickness, corneal volume, or anterior corneal curvature [11]. It provides relevant indicators of biomechanical dysfunction in keratoconus corneas. In a study including 12 normal corneas and 12 keratoconus corneas, a significant difference was found in Deformation Amplitude between normal and keratoconus corneas after adjusting for age, corneal central thickness, and intraocular pressure [12]. In experimental settings, the cross-linking of rabbit corneas results in a significant decrease in the Peak Distance and Deformation Amplitude assessed using Corvis ST [13]. The same findings have recently been reported in a clinical study [14].

The limitation of clinical studies assessing air-puff-derived corneal biomechanical indices is the absence of a reference technology that directly assesses stiffness and viscoelasticity. Enantiomorphism is a major physiological property of the normal cornea; it shows mirror symmetry, with the body median axis as the symmetry axis. This has been verified for many of its physiological parameters including its thickness and curvature [15]. Corneal enantiomorphism in normal eyes is not defined by only the mirror symmetry of the corneal anterior surface astigmatism axis, but also by the symmetry of various corneal features including the keratometry, cylinder, and thickness. Mirror symmetry is also observed when pachymetry maps of fellow eyes are compared. Interestingly, the loss of these corneal features is a hallmark of keratoconus. We hypothesize that corneal biomechanical behavior should also be enantiomorph in normal corneas—as suggested by the mass distribution of preferentially aligned fibrils in the cornea, which appear to exhibit a degree of midline symmetry between the left and right eyes [16]. Conversely, keratoconus corneas feature a loss of corneal enantiomorphism as a major diagnosis indicator [17]. Asymmetry of corneal biomechanics should also characterize keratoconus corneas.

In the present study, we assessed the reproducibility and diagnosis value of Corvis ST indices in normal and keratoconus corneas, considering their ability to distinguish keratoconus from normal corneas and to demonstrate the expected enantiomorphism features of both corneal conditions.

## 2. Patients and Methods

We retrospectively selected patients with either mild to advanced keratoconus or normal corneas from a prospective observational cohort of patients (CCK-CONE) registered in the Health Data Hub and approved by the Institut National des Données de Santé (#255645). All patients gave informed consent. All procedures followed the tenets of the declaration of Helsinki.

All eyes were assessed with Corvis ST (Oculus Optikgeräte GmbH, Wetzlar, Germany), specular corneal topography (MS39; CSO, Firenze, Italy), and spectral-domain optical coherence tomography (OCT; RTVue; Optovue, Inc., Fremont, CA, USA). The main outcome measures were the 15 Corvis ST indices.

The inclusion criteria for the keratoconus group were the following: a positive keratoconus diagnosis based on slit-lamp examination, specular topography and spectral-domain OCT, the absence of a history of ocular surgery (including collagen cross-linking, intra corneal ring segment implantation, and keratoplasty) or trauma, the absence of associated corneal pathologic features, and the absence of contact lens wear during the 3 weeks preceding the assessment. The clinical features of keratoconus include corneal ectasia, apical thinning, Vogt striae, the Fleischer ring, the Munson sign, the Rizutti sign, or corneal scarring consistent with keratoconus. The diagnosis of keratoconus was facilitated by the use of corneal topographic data: a keratoconus appearance on the topography (skewed asymmetric bowtie, central or inferior steep zone, or claw shape), positive topographic indices (mean keratometry >47 diopters, or inferior–superior value >1.4 diopters in the central 3.0 mm). We did not use the grade of severity to analyze subgroups, but all stages of severity were included except forme fruste keratoconus. The presence of epithelial thinning in the thinnest corneal zone or a doughnut pattern on the SD-OCT epithelial map, and positive keratoconus OCT classification on OCT scans including the conus, were used as SD-OCT indicators of keratoconus [18,19]. The keratoconus group included 55 eyes from 32 patients.

The inclusion criteria for the control group were the following: normal topography, negative topography indices (K ≤ 47 D and I-S ≤ 1.4), SD-OCT indicators negative of keratoconus, normal SD-OCT scans, normal slit-lamp examination, no history of ocular surgery or trauma, no contact lens wear, and no history of eye diseases. Sixty-two eyes from 31 normal subjects were included in the control group.

Each Corvis ST exam consisted of three measurements for each eye. Three exams were performed by three orthoptists for each patient to assess reproducibility.

Inter-observer reproducibility was assessed with the coefficient of variation of the three measurements. Friedman’s analysis of variance was used to assess inter-observer differences. Differences between fellow eyes and between groups were assessed with non-parametric tests (Wilcoxon paired test and Mann–Whitney U test). The Spearman rank correlation coefficient was used to assess the correlations between indices and corneal thickness and intraocular pressure. To compare the Corvis ST indices for their ability to distinguish keratoconus from normal corneas, we calculated their sensitivity (i.e., true positive/(true positive + false negative) and accuracy (i.e., (true positive + true negative)/total number of observations) using a specificity (i.e., true negative/(true negative + false positive) set at 95 ± 1% (i.e., diagnosis thresholds = 5th or 95th percentiles of the indices in the control group). Analyses were performed using a software program (Statistica version 6.1; StatSoft France, Maisons-Alfort, France). As the location of the cone may affect the deformation following the air puff, and therefore the outcomes, the results were stratified according to the location of the thinnest point.

## 3. Results

One hundred and seventeen eyes were included in the study. Patients were assessed between January 2022 and July 2022. Keratoconus patients featured a mean age of 34.2 ± 14.2 years (mean ± standard deviation). In nine patients, the Corvis ST analysis was unsuccessful in the most-affected eye due to severe corneal ectasia. Normal subjects featured a mean age of 23.2 ± 2.6 years. Keratoconus patients were significantly older than controls (*p* < 0.00001).

Table 1 shows the Corvis ST indices assessed in both groups.

### 3.1. Control Group

**Figure 1:**
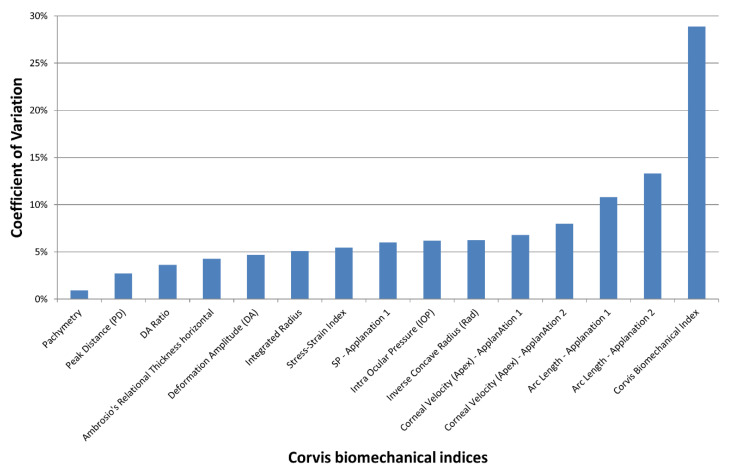
Mean coefficient of variation (COV) of the Corvis ST indices obtained by 3 different orthoptists in the control group. The inter-observer reproducibility decreases with increased COV.

In the control group, Friedman’s analysis of variance showed no significant differences between the three observers for all indices (*p* > 0.1). The lowest coefficient of variation (COV) of the Corvis indices (i.e., best reproducibility) was obtained with Pachymetry and the highest (weakest reproducibility) with the Corvis Biomechanical Index (CBI; Figure 1). The mean Corvis Biomechanical Index was 28.9%. All indices except the CBI and arc length—Applanation 1 and 2—featured a COV of less than 10%. The COV of the SSI (Stress Strain Index) and PD (Peak Distance) were, respectively, 5.5% and 2.7%. 

All the Corvis ST indices except the Peak Distance and Strain Stress index significantly correlated with Pachymetry (Table 1). All the Corvis ST indices except the inverse concave Radius significantly correlated with intraocular pressure (Table 1).

Corneal enantiomorphism was confirmed for all the Corvis ST indices except IOP—which was significantly higher in the right eye compared with the left eye—and the Corvis Biomechanical Index, which was significantly lower in the right eye compared with the left eye (Table 2). The comparison of the thinnest corneas with the thickest showed no significant differences for any of the Corvis ST indices except pachymetry, as expected (Table 2).

### 3.2. Keratoconus Group

**Figure 2:**
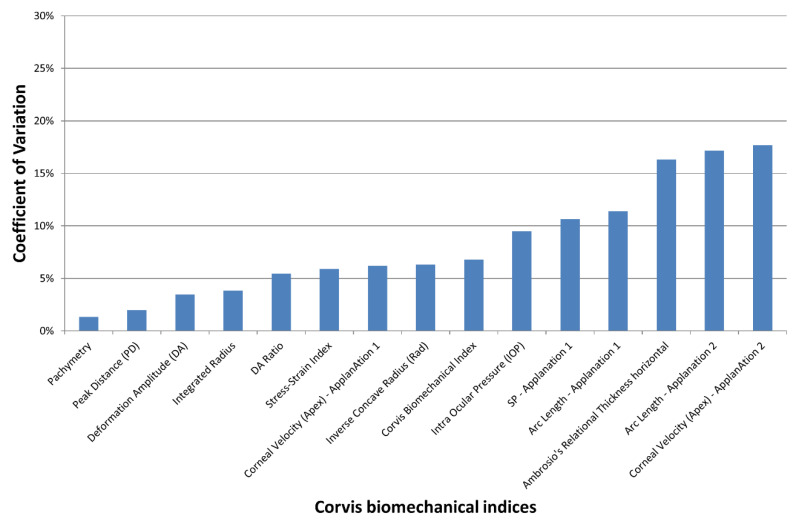
Mean coefficient of variation (COV) of the Corvis ST indices obtained by 3 different orthoptists in the keratoconus group. The inter-observer reproducibility decreases with increased COV.

In the keratoconus group, Friedman’s analysis of variance showed no significant differences between the three observers for all indices (*p* > 0.1). The lowest coefficient of variation of the Corvis ST indices (i.e., best reproducibility) was obtained with Pachymetry and the highest (weakest reproducibility) with the Corneal Velocity (apex)—Applanation 2 (Figure 2). All indices except the Corneal Velocity (Apex)—Applanation 2; the Stiffness Parameter—Applanation 1; the Arc Length—Applanation 1 and 2; and Ambrosio’s Relational Thickness horizontal featured a COV of less than 10%. The COV of the SSI was 5.9%.

All the Corvis ST indices except the Peak Distance significantly correlated with Pachymetry (Table 1). All the Corvis ST indices except the Arc Length—Applanation 1 and 2 and Corneal Velocity (Apex)—Applanation 1 significantly correlated with intraocular pressure (Table 1).

For all keratoconus patients, the most-affected cornea (i.e., the steepest cornea on the specular corneal topography) was the thinnest one, as assessed with Corvis ST. The loss of corneal enantiomorphism along with more altered biomechanical indices in the thinnest cornea was confirmed for all the Corvis ST indices except the Arc Length—Applanation 1 and 2; the Corneal Velocity (Apex)—Applanation 1 and 2; and the Peak Distance.

Among the indices independent of corneal thickness in the control group, the SSI featured unique properties (high reproducibility, expected enantiomorphism characteristics in both groups), making it a relevant biomarker of corneal biomechanical behavior.

### 3.3. Comparison of Keratoconus with Control Corneas

**Figure 3:**
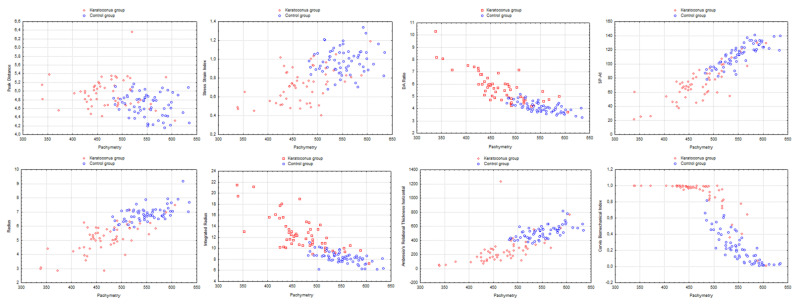
Scatterplots of the most relevant Corvis ST indices as a function of Pachymetry in both groups.

Significant differences between both groups, with more altered biomechanical indices in the keratoconus group, were found for all the Corvis ST indices (Table 1). A posteriori power analysis showed that this figure was at least 80% (i.e., β < 20%) for all indices and at least 90% for 11 out of 15 indices. As most parameters were dependent on Pachymetry and intraocular pressure, we assessed differences after the indices were divided by pachymetry or intraocular pressure. The differences were still present after adjustment for pachymetry and intraocular pressure for all indices except the Arc Length—Applanation 1. Figure 3 shows the scatterplots of the most relevant indices as a function of Pachymetry in both groups. Graphically, the Corvis Biomechanical Index featured the best ability to distinguish keratoconus from normal corneas independently of Pachymetry.

To compare the Corvis ST indices for their ability to distinguish keratoconus from normal corneas, we calculated their sensitivity and accuracy using either the 5th or 95th percentiles of the indices in the control group as diagnosis thresholds (i.e., specificity set at 95 ± 1% for all indices; Table 3). The best accuracy and sensitivity were obtained with the Corvis Biomechanical Index (accuracy, 92%; sensitivity, 91%). The weakest accuracy and sensitivity were obtained with the Peak Distance (accuracy, 66%; sensitivity, 33%).

### 3.4. Stratified Analysis

Thirty-one keratoconi featured a thinnest point that was less than 1 mm from the corneal apex on the vertical axis, as measured with SD-OCT (central keratoconus), and 24 featured a thinnest point that was more than 1 mm from the corneal apex (peripheral keratoconus). No significant differences between central and peripheral keratoconus were observed for any of the Corvis ST indices (Table 4).

Keratoconus corneas with their thinnest point at less than 1 mm from the corneal apex on the vertical meridian were classified as central. Keratoconus corneas with their thinnest point farther than 1 mm from the corneal apex on the vertical meridian were classified as peripheral.

## 4. Discussion

In the present study, most standard Corvis ST indices and indices provided by the Vinciguerra screening report featured good repeatability, as previously demonstrated for standard and recent indices [20,21] The exam could be performed in all normal corneas, but 9 out of 55 keratoconus corneas could not be assessed due to advanced keratoconus.

Among the 15 indices provided by the Corvis ST device, we found the Corvis Biomechanical Index (CBI) to be the most accurate in distinguishing keratoconi from normal corneas. The CBI featured high reproducibility in keratoconus corneas, as previously reported [22]. We did not find studies specifically addressing the influence of tear film on the reproducibility of Corvis data; however, the use of an ultra-high-speed Scheimpflug camera should allow this device to not be dependent on tear film quality [11]—it can still differentiate keratoconi from normal corneas after adjusting for pachymetry and IOP. Although linked to pachymetry, its performance appears to be highly independent of pachymetry, as assessed graphically. This result was expected, as the CBI was developed for improving keratoconus diagnosis by combining several indices including the DA, velocity, ARTh, and a stiffness parameter [23,24] Whereas standard indices of the Corvis ST device cannot readily be used for the diagnosis of keratoconus or to demonstrate the effect of CXL in vivo, indices developed in the Vinciguerra screening report—including CBI—allow for a more accurate keratoconus diagnosis compared with standard indices [11,25]. Moreover, the diagnostic ability of the CBI is comparable to that of the Pentacam indices for differentiating normal eyes and eyes with forme fruste keratoconus [26]. Recently, a modified linear term of the Corvis Biomechanical Index (i.e., Corvis Biomechanical Factor) has been developed for improving the ABCD keratoconus classification [27]. The CBF correlates with keratoconus severity [28].

Whereas the CBI is a strong biomarker of keratoconus, it may be a weaker indicator of corneal biomechanical behavior. Its reproducibility in the control group was weak, with a 28.9% coefficient of variation between the three different observers, which makes it a poor indicator of changes in biomechanics in a given cornea over time. It was also strongly correlated (rs = −0.86) to pachymetry in the control group. Corneal stiffness increases with thickness; the biomechanical behavior of any tissue is influenced by its thickness. However, assessing corneal biomechanical properties independently of corneal thickness is an unmet need which, at least conceptually, laser interferometry should significantly help with. For instance, two corneas featuring the same central corneal thickness may have different stiffnesses and different risks of developing ectasia after refractive surgery. The effect of a given photoablation procedure on these corneas and the resulting change in refractive power may be different. Lastly, significant differences in the CBI between the left and right eyes were observed in the control group, which is a weakness of this index.

Among the Corvis ST indices, the stress-strain index (SSI) presented several interesting features, making it a valuable index for assessing corneal stiffness. The SSI is a stiffness parameter, derived from an algorithm, that showed no significant correlation with either central corneal thickness or intraocular pressure but was significantly correlated with age in the dataset used for its production [29]. First, its reproducibility was good, with a coefficient of variation between three different observers of less than 6%. This means that a 10% change in the SSI should be considered significant. Second, it was not significantly correlated with pachymetry in the control group, showing that it could assess corneal stiffness independently of corneal thickness. Lastly, the SSI featured the expected enantiomorphism properties, i.e., normal corneas were enantiomorph and keratoconus corneas were not. Compared with the CBI, the SSI’s accuracy for diagnosing keratoconus (77%) was lower. However, keratoconus is a localized corneal disorder featuring decreased stiffness in the conus area, with normal stiffness outside the conus. As Corvis ST provides an assessment of the whole cornea’s biomechanical behavior, it may be less accurate for diagnosing keratoconus than a method that provides a localized assessment, such as Brillouin microscopy or OCT-elastography [30,31,32]. Global biomechanical assessments may be more accurate when combined with structural and topographical parameters. Pentacam HR and Corvis ST parameters have been combined and analyzed using a random forest method to improve corneal ectasia detection, with very high sensitivity/specificity—even in forme fruste keratoconus corneas (91% sensitivity, 96% specificity) [33,34,35]. Back propagation neural networks have been used to differentiate form fruste keratoconus from normal thin corneas with a 91% accuracy [36]. A recent study has demonstrated a progressive decrease in the SSI with more advanced keratoconus stages, while the SSI was relatively independent of IOP and corneal central thickness but positively correlated with age [37]. A recent study showed that in eyes with ocular hypertension, the SSI decreases after initiation of treatment with topical antiglaucoma medications [38]. Whether this decrease is related to decreased intraocular pressure or drug-related ocular surface damage is still to be determined. 

We found the intraocular pressure to not be enantiomorph in the control group, which was unexpected. However, as the Corvis ST exam was performed in the right eye first, we can hypothesize that the first eye exam influenced the fellow-eye exam.

The study’s limitations include the absence of a successful Corvis ST examination in 9 out of 55 keratoconus corneas, the absence of a reference measure of corneal stiffness, the absence of a keratoconus subgroup analysis, and the difference in age between normal and keratoconus corneas. As corneal stiffness increases with age, comparing older keratoconus patients with younger controls should result in weaker differences between both groups. Despite this age difference, all indices were significantly impaired in the keratoconus group. Whereas we lack a reference technology to directly measure corneal stiffness in vivo—as can be done in an experimental setting with, for instance, extensometry or OCT-elastography—we have used corneal enantiomorphism in addition to repeatability and the ability to distinguish keratoconus from normal corneas as clinical indicators. This led us to select the SSI as the most relevant index for assessing corneal stiffness and the CBI as the most efficient index for diagnosing keratoconus, using a dataset different from those used to develop these indices. These findings are in good agreement with previous studies [19,22,25,33]. As our objective was not to determine the best threshold values for the indices but to compare them with each other, we did not use receiver operating characteristic (ROC) curves, but the 5th and 95th percentiles measured in the control group as threshold values to set the specificity at 95 ± 1%. The absence of a successful Corvis ST examination in severe keratoconus is probably a limitation of the technology itself. However, assessing corneal biomechanics in advanced keratoconus may not be mandatory, as the diagnosis is clinically evident, the corneal condition well-known, and the indication for transplantation is evident.

**In conclusions:** The CBI was the best index for keratoconus diagnosis amid the Corvis ST indices. As a consequence, it could be considered a relevant supplementary biomarker for screening candidates for refractive surgery if combined with corneal topography and optical coherence tomography. Conversely, relying on this Corvis biomarker alone is not advisable, as the performance would be less than that of the other two technologies. In addition, CBI reproducibility was weak in normal corneas and was strongly dependent on corneal thickness. Conversely, the SSI was independent of corneal thickness, highly reproducible, and provided the expected enantiomorphism characteristics in normal and keratoconus corneas—making it a very acceptable biomarker of corneal biomechanical behavior. The SSI may be an interesting index for assessing changes in corneal stiffness over time or changes induced by corneal refractive surgery or cross-linking.

## Figures and Tables

**Table 1:** Corvis indices in normal and keratoconus corneas.

Corvis Index	Group	N	Mean	Median	Minimum	Maximum	5th Percentile	95th Percentile	Standard Deviation	Mean COV	Correlation with Pachymetry (rs)	Mean per 100 µm-CCT	Correlation with IOP (rs)	Mean per 10-mm Hg IOP
Arc Length–Applanation 1	Control	62	2.27	2.28	1.68	2.84	1.84	2.66	0.27	10.8%	0.49	0.41	0.58	1.30
	Keratoconus	55	1.90	1.85	1.35	2.46	1.47	2.43	0.29	11.4%	0.40	0.41	0.26	1.34
*p (KC versus control)*			*0.000000*							*0.70*		*0.73*		*0.40*
Arc Length–Applanation 2	Control	62	2.07	1.99	1.56	2.89	1.67	2.69	0.32	13.3%	0.56	0.38	0.39	1.19
	Keratoconus	55	1.54	1.51	1.01	2.16	1.08	2.04	0.29	17.2%	0.43	0.33	0.23	1.08
*p (KC versus control)*			*0.000000*							*0.07*		*0.000003*		*0.01*
Corneal Velocity (Apex)–Applanation 1	Control	62	0.15	0.15	0.11	0.20	0.12	0.18	0.02	6.8%	−0.50	0.03	−0.84	0.09
	Keratoconus	55	0.17	0.17	0.11	0.22	0.13	0.22	0.03	6.2%	−0.53	0.04	−0.36	0.12
*p (KC versus control)*			*0.000000*							*0.54*		*0.000000*		*0.000000*
Corneal Velocity (Apex)–Applanation 2	Control	62	−0.26	−0.26	−0.32	−0.20	−0.30	−0.21	0.03	8.0%	0.27	−0.05	0.65	−0.15
	Keratoconus	55	−0.88	−0.31	−11.90	−0.18	−8.52	−0.22	2.43	17.7%	0.52	−0.20	0.46	−0.63
*p (KC versus control)*			*0.04*							0.07		*0.04*		*0.03*
Intra Ocular Pressure (IOP. mm Hg)	Control	62	17.5	17.0	13.2	22.7	14.5	21.5	2.1	6.2%	0.39	3.2		
	Keratoconus	55	14.76	14.33	7.25	31.83	10.00	19.33	3.57	9.5%	0.35	3.18		
*p (KC versus control)*			*0.000001*							*0.14*		*0.97*		
Peak Distance (PD)	Control	62	4.68	4.73	4.15	5.16	4.23	5.08	0.27	2.7%	−0.20	0.85	−0.75	2.72
	Keratoconus	55	4.95	4.95	4.32	6.37	4.48	5.36	0.33	2.0%	−0.04	1.07	−0.42	3.54
*p (KC versus control)*			*0.000004*							*0.04*		*0.000000*		*0.000000*
Inverse Concave Radius (Rad)	Control	62	6.89	6.82	5.76	9.17	6.11	7.86	0.55	6.3%	0.50	1.25	0.16	3.98
	Keratoconus	55	5.18	5.24	2.86	7.48	3.00	6.99	1.02	6.3%	0.60	1.10	0.27	3.62
*p (KC versus control)*			*0.000000*							*0.93*		*0.000000*		*0.004*
Deformation Amplitude (DA)	Control	62	1.03	1.03	0.83	1.21	0.88	1.17	0.09	4.7%	−0.34	0.19	−0.75	0.60
	Keratoconus	55	1.23	1.22	0.94	1.81	1.00	1.57	0.17	3.5%	−0.57	0.27	−0.66	0.89
*p (KC versus control)*			*0.000000*							*0.02*		*0.000000*		*0.000000*
Pachymetry (µm)	Control	62	551	547	482	635	494	612	37	0.9%	1.00		0.39	
	Keratoconus	55	470	465	339	606	353	568	56	1.3%	1.00		0.35	
*p (KC versus control)*			*0.000000*							*0.09*				
Stress-Strain Index	Control	62	0.98	0.98	0.68	1.34	0.75	1.21	0.14	5.5%	0.09	0.18	0.46	0.56
	Keratoconus	55	0.73	0.74	0.40	1.19	0.47	1.03	0.18	5.9%	0.45	0.15	0.38	0.51
*p (KC versus control)*			*0.000000*							*0.52*		*0.000035*		*0.004*
DA Ratio	Control	62	4.12	4.08	3.27	5.20	3.55	4.87	0.43	3.6%	−0.67	0.75	−0.74	2.41
	Keratoconus	55	5.72	5.57	3.73	10.30	4.23	8.07	1.23	5.5%	−0.78	1.26	−0.43	4.17
*p (KC versus control)*			*0.000000*							*0.02*		*0.000000*		*0.000000*
Integrated Radius	Control	62	8.31	8.30	6.17	11.10	6.30	10.07	1.04	5.1%	−0.61	1.52	−0.69	4.86
	Keratoconus	55	12.62	11.90	7.23	21.45	9.25	19.45	3.10	3.8%	−0.66	2.78	−0.53	9.30
*p (KC versus control)*										*0.42*		*0.000000*		*0.000000*
Ambrosio’s Relational Thickness horizontal	Control	62	519	520	362	819	393	652	94	4.3%	0.63	94	0.29	299
	Keratoconus	55	261	232	42	1235	54	577	191	16.3%	0.73	53.57	0.39	175.52
*p (KC versus control)*			*0.000000*							*0.0004*		*0.000000*		*0.000000*
Stiffness Parameter—Applanation 1	Control	62	113	114	78	141	87	137	16	6.0%	0.86	20	0.64	65
	Keratoconus	55	73	71	22	130	26	122	24	10.6%	0.73	15.21	0.56	49.85
*p (KC versus control)*										*0.04*		*0.000000*		*0.000000*
Corvis Biomechanical Index	Control	62	0.24	0.21	0.01	0.82	0.02	0.60	0.20	28.9%	−0.86	0.05	−0.42	0.15
	Keratoconus	55	0.88	0.98	0.01	1.00	0.36	1.00	0.22	6.8%	−0.80	0.19	−0.47	0.64
*p (KC versus control)*			*0.000000*							*0.000000*		*0.000000*		*0.000000*

The brown color shows significant differences betwween keratoconus and control corneas. The blue color shows absence of correlation (*p* > 0.05) of the indices (i.e., independence) with pachymetry or intraocular pressure.

**Table 2:** Biomechanical enantiomorphism analysis in normal and keratoconus corneas.

Corvis Indices	Group	N	Right Eye	Left Eye	*p*	Thinnest Cornea	Thickest Cornea	*p*
Arc Length—Applanation 1	Control	31	2.31	2.23	*0.42*	2.26	2.27	*0.88*
	Keratoconus	23	1.79	1.95	*0.00003*	1.82	1.88	*0.35*
Arc Length—Applanation 2	Control	31	2.08	2.06	*0.51*	2.02	2.13	*0.06*
	Keratoconus	23	1.52	1.56	*0.00003*	1.46	1.61	*0.19*
Corneal Velocity (Apex)—Applanation 1	Control	31	0.15	0.15	*0.22*	0.15	0.15	*0.52*
	Keratoconus	23	0.18	0.17	*0.02*	0.18	0.18	*0.47*
Corneal Velocity (Apex)—Applanation 2	Control	31	−0.25	−0.26	*0.94*	−0.25	−0.26	*0.82*
	Keratoconus	23	−0.83	−1.16	*0.18*	−0.85	−1.21	*0.35*
Intra Ocular Pressure (IOP. mm Hg)	Control	31	17.9	17.2	*0.009*	17.6	17.5	*0.61*
	Keratoconus	23	14.1	14.4	*0.82*	14.0	14.5	*0.31*
Peak Distance (PD)	Control	31	4.67	4.70	*0.39*	4.65	4.71	*0.05*
	Keratoconus	23	4.92	4.90	*0.95*	4.89	4.94	*0.31*
Inverse Concave Radius (Rad)	Control	31	6.81	6.96	*0.17*	6.85	6.92	*0.42*
	Keratoconus	23	4.83	5.36	*0.03*	4.68	5.43	*0.002*
Deformation Amplitude (DA)	Control	31	1.03	1.02	*0.83*	1.03	1.02	*0.59*
	Keratoconus	23	1.28	1.20	*0.03*	1.29	1.21	*0.03*
Pachymetry (µm)	Control	31	551	551	*0.84*	547	555	*0.000001*
	Keratoconus	23	454	481	*0.09*	444	489	*0.00004*
Stress-Strain Index	Control	31	0.98	0.98	*0.42*	0.99	0.97	*0.09*
	Keratoconus	23	0.68	0.75	*0.03*	0.66	0.75	*0.04*
DA Ratio	Control	31	4.09	4.15	*0.16*	4.12	4.13	*0.64*
	Keratoconus	23	6.09	5.57	*0.18*	6.31	5.48	*0.004*
Integrated Radius	Control	31	8.23	8.39	*0.25*	8.30	8.31	*0.90*
	Keratoconus	23	13.82	11.97	*0.02*	14.19	11.85	*0.002*
Ambrosio’s Relational Thickness horizontal	Control	31	532	505	*0.007*	514	524	*0.41*
	Keratoconus	23	262	253	*0.45*	179	334	*0.0002*
Stiffness Parameter—Applanation 1	Control	31	112	114	*0.22*	113	113	*0.67*
	Keratoconus	23	67	78	*0.05*	63	80	*0.0002*
Corvis Biomechanical Index	Control	31	0.21	0.27	*0.003*	0.25	0.23	*0.37*
	Keratoconus	23	0.90	0.88	*0.96*	0.96	0.82	*0.002*

An expected significant difference (i.e., loss of enanthiomorphism or thinnest cornea showing more altered indices in the keratoconus group) is highlighted with the brown color. An unexpected lack of significant difference (i.e., maintained enanthiomorphism or absence of difference between thinnest and thickest corneas in the keratoconus group) or unexpected presence of significant difference (i.e., loss of enanthiomorphism or thinnest cornea showing more altered indices in the control group) is highlighted with the blue color.

**Table 3:** Assessment of Corvis indices for the diagnosis of keratoconus.

Corvis Indices	Threshold Value	Specificity	Sensitivity	Accuracy (Percentage of True Positive and True Negative Observations)
Arc Length—Applanation 1	<1.84	95%	49%	74%
Arc Length—Applanation 2	<1.67	94%	71%	83%
Corneal Velocity (Apex)—Applanation 1	>0.18	96%	33%	67%
Corneal Velocity (Apex)—Applanation 2	<−0.30	94%	58%	77%
Intra Ocular Pressure	<14.5 mm Hg	95%	51%	74%
Peak Distance	>5.08	95%	33%	66%
Inverse Concave Radius	<6.11	95%	84%	90%
Deformation Amplitude	>1.17	95%	62%	79%
Pachymetry	<494 µm	94%	73%	84%
Stress-Strain Index	<0.75	95%	56%	77%
DA Ratio	>4.87	95%	71%	84%
Integrated Radius	>10.07	95%	82%	89%
Ambrosio’s Relational Thickness horizontal	<393	95%	87%	91%
Stiffness Parameter—Applanation 1	<87	95%	78%	87%
Corvis Biomechanical Index	>0.60	94%	91%	92%

**Table 4:** Analysis stratified according to the location of the thinnest point.

Corvis Indices	Group	N	Mean ± SD	Central versus Control *p*	Peripheral versus Control *p*	Peripheral versus Central *p*
Arc Length—Applanation 1	Control	62	2.27 + 0.27	<0.00001	0.00003	0.47
	Central keratoconus Peripheral keratoconus	31 24	1.88 + 0.30 1.93 + 0.28			
Arc Length—Applanation 2	Control	62	2.07 + 0.32	<0.00001	<0.00001	0,92
	Central keratoconus Peripheral keratoconus	31 24	1.54 + 0.27 1.51 + 0.31			
Corneal Velocity (Apex)—Applanation 1	Control	62	0.15 + 0.02	<0.00001	0.00004	0.81
	Central keratoconus Peripheral keratoconus	31 24	0.17 + 0.03 0.17 + 0.02			
Corneal Velocity (Apex)—Applanation 2	Control	62	−0.26 + 0.03	0.03	0.31	0.40
	Central keratoconus Peripheral keratoconus	31 24	−1.05 + 2.90 −0.67 + 1.67			
Intra Ocular Pressure (IOP. mm Hg)	Control	62	17.5 + 2.1	0.00003	0.0001	0.98
	Central keratoconus Peripheral keratoconus	31 24	14.7 + 2.6 14.8 + 4.6			
Peak Distance (PD)	Control	62	4.68 + 0.27	0.0004	0.00005	0.44
	Central keratoconus Peripheral keratoconus	31 24	4.92 + 0.37 4.999 + 0.29			
Inverse Concave Radius (Rad)	Control	62	6.89 + 0.55	<0.00001	<0.00001	0.61
	Central keratoconus Peripheral keratoconus	31 24	5.23 + 1.12 5.12 + 0.88			
Deformation Amplitude (DA)	Control	62	1.03 + 0.09	<0.00001	<0.00001	0.18
	Central keratoconus Peripheral keratoconus	31 24	1.20 + 0.17 1.25 + 0.18			
Pachymetry (µm)	Control	62	551 + 37	<0.00001	<0.00001	0.53
	Central keratoconus Peripheral keratoconus	31 24	473 + 61 466 + 49			
Stress-Strain Index	Control	62	0.98 + 0.14	<0.00001	<0.00001	0.71
	Central keratoconus Peripheral keratoconus	31 24	0.73 + 0.19 0.72 + 0.18			
DA Ratio	Control	62	4.12 + 0.43	<0.00001	<0.00001	0.57
	Central keratoconus Peripheral keratoconus	31 24	5.65 + 1.14 5.80 + 1.34			
Integrated Radius	Control	62	8.31 + 1.04	<0.00001	<0.00001	0.71
	Central keratoconus Peripheral keratoconus	31 24	12.72 + 3.36 12.49 + 2.78			
Ambrosio’s Relational Thickness horizontal	Control	62	519 + 94	<0.00001	<0.00001	0.92
	Central keratoconus Peripheral keratoconus	31 24	262 + 160 258 + 229			
Stiffness Parameter—Applanation 1	Control	62	113 + 16	<0.00001	<0.00001	0.41
	Central keratoconus Peripheral keratoconus	31 24	74 + 26 70 + 23			
Corvis Biomechanical Index	Control	62	0.24 + 0.20	<0.00001	<0.00001	0.09
	Central keratoconus Peripheral keratoconus	31 24	0.84 + 0.27 0.94 + 0.11			

## Data Availability

Data are available upon reasonable request.
